# Linking grassland yield and nitrogen nutrition status to management practices: Towards large-scale grassland use intensity assessment

**DOI:** 10.1016/j.rsase.2025.101754

**Published:** 2025-11

**Authors:** Mathilde De Vroey, Julien Radoux, Arnaud Farinelle, Pierre Defourny

**Affiliations:** a Earth and Life Institute, Université catholique de Louvain, 2 Croix du Sud bte L7.05.16, 1348 Louvain-la-Neuve, Belgium; b Fourrages mieux, Michamps, 3 Horritine, 6600 Bastogne, Belgium

**Keywords:** Grasslands, Yield, Nitrogen, Management, AECM, Sentinel-2

## Abstract

Adapting grassland management to meet the rising demand in food production, while sustaining their ecological value, is a challenge. This highlights the need to monitor grassland use intensity to support sustainable agricultural policies. The objectives of this study are to (1) retrieve grassland yield and nitrogen content from satellite imagery and (2) link these to management practices and pedo-climatic conditions. A stepwise linear regression performed best out of 7 different methods to retrieve grasslands dry matter yield (DM, t/ha), nitrogen concentration (NC, g/kg) and canopy nitrogen content (CNC, kg/ha) from Sentinel-2 reflectances and indices. The models are calibrated using field measurements made during three growing seasons. They are validated on an independent field dataset with a normalized RMSE of 9.5%, 15.6%, and 6.7% for DM, NC, and CNC respectively. In addition, the nitrogen nutrition index (NNI) is computed from the estimated DM and NC to evaluate grasslands nitrogen nutrition status. The retrieval models are then combined with grassland classification and mowing detection methods developed in previous studies to evaluate grasslands vegetation status in spring and harvested forage yield at regional scale. These large-scale experiments consistently showed the impacts of management practices and pedo-climatic conditions on grassland yield and nitrogen content.

## Highlights

• Grassland biophysical variables estimated from Sentinel-2 data.
• Linear model performs best out of 7 methods.
• Remote sensing identifies relationships between inputs and outputs of Grassland Use Intensity.
• Differences between agro-ecological regions are retrieved without ancillary information.

## 1 Introduction

### 1.1 Background and conceptual framework

Grasslands cover 20%–47% of the global ice-free land surface, depending on definitions (Piipponen et al., 2022). They are a key element of many agricultural systems as they provide nearly half of the feed requirements for global livestock production (O’Mara, 2012, Herrero et al., 2013). Beyond food production, they deliver key ecosystem services, including carbon sequestration, water storage, and biodiversity support (Bengtsson et al., 2019, Chang et al., 2021, O’Mara, 2012, Pärtel et al., 2005, Zeller et al., 2017). However, agricultural intensification increasingly threatens their ecological condition (O’Mara, 2012, Silva et al., 2008). Balancing grassland management to sustain ecosystem services while meeting rising demands for dairy and meat production is a major challenge, highlighting the need to monitor grassland use intensity (GUI) at adequate spatial and temporal scales to support sustainable agricultural policies.

Most studies on GUI focus on management practices, i.e. grazing, mowing, and fertilization (Blüthgen et al., 2012, Peeters et al., 2014, Tonn et al., 2020, Lange et al., 2022). However, these practices have site-specific impacts that depend on agro-pedo-climatic conditions (Grigulis et al., 2013, Abdalla et al., 2018). Therefore, management-based indicators alone are insufficient for GUI monitoring.

We propose a framework tailored for GUI measurement, integrating the three dimensions of land use intensity as outlined by Erb et al. (2013): (i) inputs, including mowing, grazing, and fertilization; (ii) outputs, such as forage yield and quality; and (iii) outcomes, evaluating biodiversity and ecosystem services. This framework allows for assessing synergies and trade-offs between production outputs and unintended ecological impacts while linking management practices to ecological effects.

Although they are all important to consider for GUI analysis, not all aspects are directly addressed here. The characterization of grasslands in terms of management practices was addressed in De Vroey et al. (2022b). In this study, the focus is set on outputs, and more specifically dry matter yield (DM), nitrogen (N) content and the nitrogen nutrition index (NNI).

Nitrogen plays a central role in plant growth and quality, influencing processes such as carbon fixation and light harvesting (Berger et al., 2020b). The NNI is a widely used indicator of nitrogen saturation in plants and is derived from DM and N content measurements. It is based on nitrogen dilution curves, which describe the decrease in N uptake with increasing biomass during the vegetative growth stage (Gastal and Lemaire, 2002). The NNI provides insights into the nutritional status of grasslands and serves as a tool for fertilization management.

### 1.2 Remote sensing of grassland yield and nitrogen content

Remote sensing offers a scalable alternative to traditional ground-based methods for monitoring grassland DM and N content (Ali et al., 2016a, Reinermann et al., 2020).

Many studies use empirical approaches, such as regression models, to directly link spectral reflectance data to target variables without prior assumptions. Regression-based studies have retrieved grassland DM with $R^{2}$ ranging from 0.68, using SPOT imagery to map DM in a watershed in France (Dusseux et al., 2015), to 0.81, using simulated Sentinel-2 (S2) bands on experimental plots in South Africa (Sibanda et al., 2015).

More recent studies have applied deterministic approaches (e.g. radiative transfer models (RTM)) relying on physical principles to simulate plant processes and retrieve primary variables, such as leaf area index (LAI) and chlorophyll content. These primary variables can then be linked to secondary variables, including DM and N content (Weiss et al., 2020). RTM-based studies for grassland DM retrieval have typically been conducted on relatively small areas, ranging from single test plots (Schwieder et al., 2020) to a few parcels (Boegh et al., 2013, Punalekar et al., 2018, Cisneros et al., 2020). While deterministic models achieve relatively good results for LAI retrieval ($R^{2}$ 0.57–0.83), they often report poorer performance for DM retrieval ($R^{2}$ 0.22–0.54).

Compared to DM, relatively few studies have explored the retrieval of N content in grasslands, and most have relied on field spectroscopy and small test sites (Boegh et al., 2013, Adjorlolo et al., 2014). Recently, Cisneros et al. (2020) used field spectroscopy to simulate S2 bands and retrieved N content on experimental plots with an RMSE of 3.4 g/kg. Pullanagari et al. (2021) retrieved grassland N content with a normalized RMSE of 14% through a convolutional neural network based on field spectroscopy.

These studies retrieve N concentration (NC, i.e. in g of N per kg of dry matter). From a radiative transfer point of view, however, Canopy Nitrogen Content (CNC, i.e. g of N per land area) should be better related to vegetation reflectances as the total quantity of N in the canopy influences the electromagnetic signals.

In optical remote sensing, LAI and DM are primarily associated with reflectances in the red edge and near-infrared wavelength domains (Delloye et al., 2018, Hank et al., 2019), while chlorophyll and N content are related to reflectances in the visible and red edge wavelength domains (Hank et al., 2019).

Overall, while studies on grassland biophysical variable retrieval show promising results, further research is needed to improve the robustness and transferability of models. Testing these approaches over larger areas and longer time periods is essential to enable reliable monitoring of productivity and link it to agroenvironmental contexts and management practices.

### 1.3 Scope and objectives

The objectives of this study are to (1) retrieve grassland yield and nitrogen content from satellite imagery and (2) link these to management practices and pedo-climatic conditions.

More specifically, the first objective is to retrieve grasslands dry matter yield (DM, t/ha), nitrogen concentration (NC, g/kg), canopy nitrogen content (CNC, kg/ha), and nitrogen nutrition index (NNI) from Sentinel-2 (S2) multi-spectral reflectances. Different regression models were calibrated on S2 bands and indices, averaged per parcel, to retrieve the biophysical variables: ordinary and ridge linear regressions (McDonald, 2009), random forest regressions, sequential neural network regression and support vector regression.

To achieve the second objective, we integrate the biophysical variable retrieval models with EO-based mowing detection and grassland classification approaches. Through this integrated approach, we evaluate differences in yield and nitrogen content between intensively managed grasslands and grasslands subject to agri-environmental and climatic measures. This analysis is carried out across multiple agro-ecological regions to capture regional variations in GUI.

## 2 Data and method

To retrieve grasslands DM, NC, and CNC from S2 reflectances, three object-based regression models are built. These empirical models are cross-calibrated and validated based on field measurements from three consecutive growing seasons (2016–2018). They are then validated on independent measurements from a fourth growing season (2020) to test their temporal transferability.

**Fig. 1:**
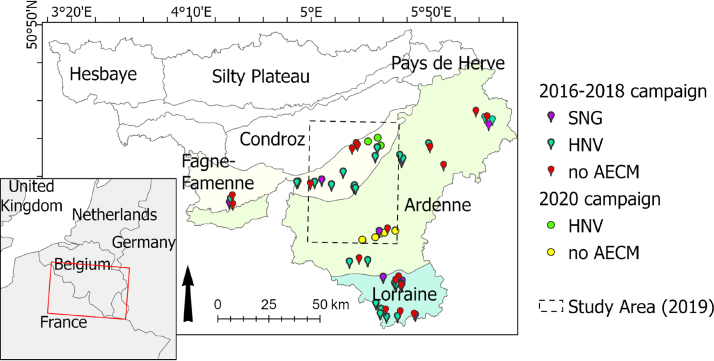
Parcels location for the field measurements during the 2016–2018 and the 2020 field campaigns, distributed across three agroecological regions of Wallonia (Belgium).

The models are then used to retrieve the vegetation status in spring and harvested forage yield of the spatially homogeneous grassland management units characterized in De Vroey et al. (2022b) over the whole study area (672.9 km^2^) for the 2019 growing season (Fig. 1).

### 2.1 Study area

The study area is located in Wallonia, the southern region of Belgium. Specifically, four agroecological regions are covered, namely Condroz, Fagne-Famenne, Ardenne and Lorraine (Fig. 1). Moving from North to South, these regions are characterized by a progressively colder climate, less productive soils, increasingly rugged topography, a higher proportion of grasslands, and a gradual decline in management intensity and grassland productivity.

Most agricultural grasslands are managed relatively intensively, with grazing, mowing, or a combination of both, starting around mid-April. In grasslands with agri-environment-climate measures (AECM) subsidized by the European Union Common Agricultural Policy (CAP), however, exploitation is only allowed during a restricted period.

The AECM aim at preserving biodiversity and protecting soil and water through more extensive management. The use of phytosanitary products is prohibited and a variety of other measures are defined by technical specifications. A first type of AECM, called “high nature value grasslands” (HNVG), are selected for their high ecological potential, and management requirements are determined for each case specifically, based on expert diagnosis. The minimum requirements state that (i) mowing is obligatory, either after July the 1st or the 15th, (ii) 10% of untouched zones must be preserved and (iii) fertilization is prohibited. Another type of AECM are “semi-natural grasslands” (SNG). This status can be given to any type of grassland and implies a fixed set of requirements (corresponding to the MB2 AECM). Fertilization is limited to one application of organic manure between June 16th and August 15th. The SNG further require (i) the obligation to mow (or graze) between June 16th and October 31st, (ii) the preservation of 5 to 10% of untouched refuge zone.

### 2.2 Field measurements

A large field survey was carried out across Wallonia, in the framework of operational in situ grassland monitoring by “Fourrages Mieux” (Farinelle, 2020). The survey aimed to evaluate the forage quality of permanent grasslands with and without AECM. The monitored grassland parcels (n = 59) include HNVG (n = 27), SNG (n = 14) and permanent grasslands without AECM (n = 18) for the sake of comparison (Fig. 1). This is expected to cover the range of observable biophysical variables, but it is not representative of the actual distribution of these classes because parcels without AECM account for more than 90% of the agricultural grassland.

During three growing seasons (2016–2018), vegetation samples were cut and measured before each mowing — or grazing — event on all monitored grasslands. The same set of 59 parcels were monitored during the whole study period. The parcels were sampled 1 to 5 times per season, resulting in a total of 353 measurements. For each measurement in each parcel, 4 samples were collected. In grasslands with AECM, the location of the 4 samples was chosen by expert on the field to be as representative as possible of each parcel. In grasslands with no AECM, 4 sample points were chosen randomly on a homogeneous area of each parcel.

For each sample, an area between 6 and 12 m^2^ was cut with a mower. The area was precisely measured for each sample. The cut grass was immediately weighted to obtain the wet matter yield. Then a sub-sample (150–300 g) was collected, kept cool, and brought to the lab for further measurements. Each sub-sample was dried at a temperature of 55 °C and weighed separately to measure the dry matter content. The parcel DM was estimated by averaging the measurements of the four sub-samples.

The NC was then measured through NIR spectrometry on a crushed composite of the four sub-samples taken for DM measurement. The measurements were performed in a certified lab, with hyperspectral NIR spectrometer from 780 to 2500 nm. The CNC was then computed as NC × DM.

Thirteen additional DM and NC measurements were made in 2020, specifically for this study, to test the transferability of the models to another growing season. On May 11th, 7 permanent grassland parcels without management restrictions, located in Ardenne were sampled and on June 25th, 6 HNVG, located in Fagne-Famenne were sampled (Fig. 1). In each parcel, a homogeneous area of at least 20 × 20 m was identified and the GPS coordinates of the center of the area were recorded. Four samples were collected inside the homogeneous area, with a minimum of 4 m between each sample. The samples were collected manually, by cutting 50 × 50 cm squares. Similarly to the previous campaign, the collected samples were dried and weighed. The DM yield of each parcel was estimated by averaging the DM measured for each sample, the NC was measured through NIR spectrometry on a blend of the four samples, and the CNC was computed from the NC and DM.

### 2.3 Satellite data

The biophysical variable retrieval methods developed in this study are based on S2 data. All S2 A and B top-of-atmosphere reflectance images covering the study area and period, covering five seasons (2016–2020), were downloaded and converted to surface reflectance using Sen2Cor v2.10 for atmospheric correction and the Fmask algorithm (Zhu and Woodcock, 2012) for cloud and cloud shadow masking.

All 10 m and 20 m resolution S2 bands (visible (BGR): B2-B4, red edge: B5-B7, near-infrared (NIR): B8, narrow NIR: B8A and short wave infrared (SWIR): B11–B12) and four spectral indices were used. First, the normalized difference vegetation index (NDVI) has often been linked to green above-ground biomass (Zhang et al., 2015, Dusseux et al., 2015). The three other indices are related to the red edge, which is both linked to biomass (Sibanda et al., 2015, Schwieder et al., 2020, Hardy et al., 2021) and vegetation chlorophyll content. The red edge chlorophyll index (CIre), the normalized difference red edge index (NDRE) and the S2 red edge position index (REP) were computed with Eqs. (1), (2), (3), respectively.

(1) $$CIre=\frac{B7}{B5}-1$$

(2) $$NDRE=\frac{B8-B5}{B8+B5}$$

(3) $$REP=705+35\times \left(\frac{B4+B7}{2}-B5\right)/\left(B6-B5\right)$$

### 2.4 Dataset preparation

Based on the field measurements, an object-based reference dataset was compiled to calibrate and validate the regression models for the retrieval of DM, NC, and CNC.

Field measurements were spatially and temporally matched with S2 multispectral data. Each field measurement was associated with a specific grassland parcel that included the sample points. These grassland parcels were derived by integrating parcel boundaries from the regional Land Parcel Identification System (LPIS) with a grassland classification that distinguishes between grazed and mown grasslands at the pixel level (De Vroey et al., 2022b). The resulting parcels are assumed to represent homogeneous grassland management units, where similar management practices are applied within each unit. We further hypothesize that the delineation of these management units remains mostly unchanged throughout the study period (2016–2020), as they are often demarcated by fences or hedges. The average S2 reflectance values and derived indices of all pixels completely within each management unit are used as input for the retrieval of biophysical variables.

The samples were not specifically collected on cloud-free S2 acquisition dates. Only 26 of the 353 measurements made in 2016–2018 could be associated with a cloud-free S2 acquisition on the same day. Therefore, the closest cloud-free S2 acquisition with a maximum interval of 9 days before the field measurement was considered useable. S2 images acquired after the field measurements could not be considered, since all samples were collected right before a mowing event. With a maximum interval of 9 days, 154 reference objects could be considered. This dataset was randomly split into a calibration dataset of 120 objects and an independent validation dataset of 34 objets.

For the field campaign of 2020, S2 acquisitions following the field measurement could be considered as well. Cloud-free S2 acquisitions were available within 4 days around each measurement. This dataset was used to validate the regression models at a different date.

### 2.5 Regression models

Different regression models were benchmarked on S2 bands and indices, averaged per parcel, to retrieve grasslands DM, NC, and CNC: stepwise and ridge linear regressions (McDonald, 2009), random forest regressions, sequential neural network regression and support vector regression.

For the linear regressions, a stepwise approach with a backward elimination approach was applied to select the most explanatory S2 variables for each biophysical variable. Initially, all S2-derived variables were included. At each step, a least squares linear model was fitted to the dataset and a p-value was computed for each input variable, indicating if the respective slope coefficient was significantly different from 0. The variable with the highest p-value (i.e. the least explanatory) was discarded, and a new model was fitted with the remaining variables. This process was repeated until all p-values dropped below a given threshold, namely 0.05 and 0.01.

Ordinary and gradient boosting random forest regression (Breiman, 2001) are tested with different maximum numbers of variables (between 25 and 100% of the full set). The number of iterations (between 50 and 500), the minimum number of samples at leave node (between 1 and 30) and the maximum depth (between 5 and 500) are optimized by grid search.

The sequential neural network regression (Ketkar, 2017) used a stack of one to 4 dense hidden layers with a normal kernel initializer and a nonlinear activation (ReLU). Each of these models were trained on 1000 epochs with a batch size of 5, using the ADAM optimizer. These parameters are commonly used in other studies (Manjula and Vijaya, 2020).

Two different types of kernels are used with the Support Vector Regression (Awad et al., 2015), namely a radial basis function (RBF) and a polynomial kernel of first (that is linear), second and third degree. The data is scaled before learning. The values of C (from 10 to $10^{5}$) and ϵ (between 0 and 20% of the value range) are optimized by grid search.

The performances of the different models and their parameters are compared through a 12-fold cross-validation approach, using the reference dataset of the 2016–2018 growing seasons. The RMSE was computed between measured and predicted biophysical variable values. In order to be able to compare the results for DM, NC, and CNC retrieval, the RMSE was normalized by the respective biophysical variable range.

### 2.6 Independent validation and temporal transferability

The models with the overall lowest RMSE were retrained on the whole calibration dataset. These models were then used to retrieve the DM, NC, and CNC of the 34 parcels left aside of the calibration dataset of the 2016–2018 growing season.

The models were then retrained on the full 2016–2018 growing season to test their transferability at another date with the use of the unseen parcels of the 2020 field campaign.

To compare the direct and indirect retrieval of NC and CNC, each N variable was also computed, based on the retrieved DM and the other N variable. The indirect NC was computed by dividing the retrieved DM by the retrieved CNC, and inversely, the indirect CNC by multiplying the retrieved NC by the retrieved DM.

In addition, the NNI was computed, from the retrieved DM and NC on one hand, and CNC on the other, to evaluate the potential of the EO-based biophysical variables to measure the nitrogen nutrition status of grasslands. The dilution curve defined for grasslands (Gastal and Lemaire, 2002) is given in Eq. (4).

(4) $$cNC=4.8\times DM^{-0.32}$$

where cNC is the critical nitrogen concentration, i.e. the nitrogen absorption capacity of the vegetation to reach maximum potential yield. The NNI is computed as the ratio between the actual NC and cNC.

### 2.7 Large scale application

The EO-based biophysical variables were used to perform a comprehensive regional analysis of GUI. To assess the impacts of management practices, the vegetation status in spring was compared between, on one side, grasslands with management practices designed to favor biodiversity (HNVG and SNG-MB2) and, on the other side, different types of management practices not declared as HNVG or SNG. Building on results of a previous study (De Vroey et al., 2022b), this analysis was performed for the 2019 growing season, over an area covering three agroecological regions of Wallonia — Condroz, Fagne-Famenne, and Ardenne (Fig. 1). The set of variable predictor was restricted to EO-based variables because most auxillary spatial variables are (i) strongly correlated with the agroecological regions and (ii) prone to overfitting (Meyer et al., 2018).

#### 2.7.1 Hierarchical characterization of grassland management

In this study, three types of management practice are considered, namely pastures, hay meadows with an early first mowing event (i.e. before June 16th, further referred to as “early mowing”), and hay meadows with a late first mowing event (i.e. on or after June 16th, further referred to as “late mowing”).

For HNVG and SNG, we assumed that the management practices are compliant with their guidelines, i.e. hay meadows with a late first mowing event and no fertilization. For other grasslands, we used the management units and mowing detections obtained by De Vroey et al. (2022b) to derive the management practices. Grassland management types were classified hierarchically. First, pastures that are exclusively grazed were differentiated from hay meadows (i.e. grasslands with at least one mowing event, including mixed practices) through a pixel-based classification of S2-derived LAI time series. The classification was then combined with the regional LPIS parcel boundaries to retrieve homogeneous management units. Finally, the grassland mowing detection method of Sen4CAP (De Vroey et al., 2022a), based on Sentinel-1 and Sentinel-2 time series, was used to further differentiate hay meadows by the timing and frequency of mowing events.

#### 2.7.2 Vegetation status in spring

First, the biophysical variable retrieval models are used to estimate DM and NC on a single date (1st of May) for all management units. The goal is to study the variability of the vegetation status in spring across grassland management practices.

The comparison is performed per agro-ecological region in order to reduce the potential impact of soil and climate conditions. Only parcels that have not yet been mown on the 1st of May are considered for the comparison. These parcels are identified using the mowing detection algorithm.

The retrieved DM and NC are used to compute the NNI and to map the nitrogen nutrition status of grassland across the study area.

#### 2.7.3 Harvested forage yield

The second large-scale analysis focuses on the harvested forage yield of hay meadows. By combining mowing detection and the biophysical variable retrieval models, DM and NC could be estimated before the first and second mowing events of hay meadow management unit in the study area.

The mowing detection method (De Vroey et al., 2022a), identifies mowing events for time intervals ($\left[t_{end}-t_{start}\right]$) of maximum 12 days, in which $t_{start}$ should correspond to the last satellite acquisition on tall grass (De Vroey et al., 2022a). For each mowing event, DM and NC were estimated at $t_{start}$ when $t_{start}$ corresponded to a S2 acquisition. Otherwise, the closest S2 cloud free acquisition date before $t_{start}$ is used. However, if the time between the cloud free S2 acquisition exceeds 12 days before $t_{end}$, the location is discarded. This constraint reduces the risk of having multiple mowing events in the same time interval while allowing sufficient DM and NC estimations.

This analysis was limited to grasslands with AECM or no AECM with a late mowing to guarantee a relatively uniform timing of mowing events.

## 3 Results

### 3.1 Field measurements

**Fig. 2:**
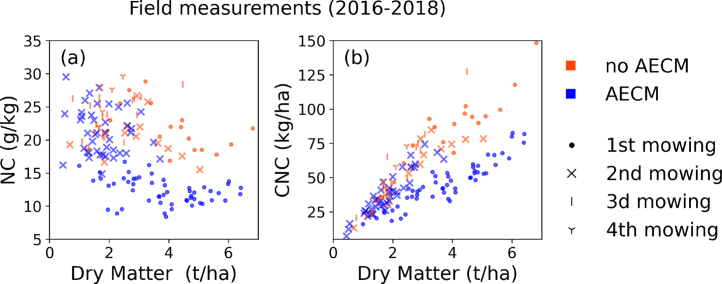
Comparison of field measurements of dry matter versus (a) N concentration (NC, g/kg), and (b) canopy N content (CNC, kg/ha) from the 2016–2018 field campaigns. The legend indicates whether or not an AECM is present and before which mowing event the measurements were made.

The field measurements of DM, NC and CNC, shown in Fig. 2, already allow to highlight some differences between grasslands with and without AECM. For example, on the first mowing event, grasslands with no AECM are characterized with higher NC than grasslands with AECM (Fig. 2(a)). At 2nd, 3d and 4th mowing event, there is no noticeable difference between AECM and no AECM.

While the measurements show no correlation between DM and NC, the CNC is strongly related to the DM, with an R^2^ of 0.81 for grasslands with AECM and 0.92 for grasslands with no AECM (Fig. 2(b)). This is expected since the CNC is computed based on the NC and the DM.

### 3.2 Variable selection and models calibration

The different models achieved satisfying results on the calibration dataset. The stepwise linear regression performed on average better than the other methods for NC and CNC. For DM, stepwise linear regression was second best to the SVR with polynomial kernel of the first degree (that is, linear kernel). Table 1 shows the results for the best hyperparameters for each method.

**Table 1:** Average root mean square error of the regression models on the calibration dataset (n = 120) with a 12-fold cross-validation.

Method	Average RMSE		
	DM (t/ha)	NC (g/kg)	CNC (kg/ha)
Stepwise linear regression	0.95	3.18	16.76
Ridge regression	0.95	3.23	17.40
SVR with polynomial kernel	0.94	3.31	16.97
SVR with RBF kernel	1.03	3.38	18.17
Gradient boosting regression	1.14	4.28	17.88
Random forest regression	1.13	4.30	17.86
NN regression	1.06	4.04	18.04

The best hyperparameters of the SVR with RBF kernel were [ϵ=0.7, C=750], [ϵ=2, C=1600] and [ϵ=4, C=2500] respectively for DM, NC and CNC; for the SVR with polynomial kernel, the first degree was better for NC and DM (respectively [ϵ=0.2, C=2000] and [ϵ=0.25, C=1000]), while the second degree was best for CNC (ϵ=1 and C=50000). The standard random forest performed best with 70% of the variables for DM and CNC while it required 80% of the variables for NC. The minimum number of samples per leaves was samll (1 for DM and NC, 3 for CNC) and the maximum depth was 5, 10 and 50 for CNC, DM and NC, respectively. The optimal percentages of features was different with the GBR, with 80% for CNC, 70% for DM and 50% for NC. GBR required larger number of leaves than RFR (10 for DM and NC, 20 for CNC) and deeper decision trees (50 for DM and NC, 100 for CNC).

**Fig. 3:**
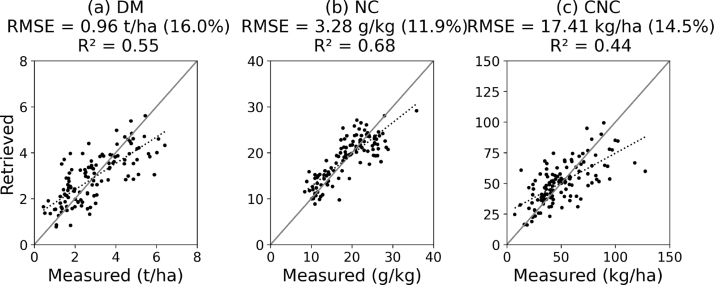
Calibration results (n=120 with leave-one-out cross validation) through stepwise multiple linear regression. (a) dry matter, (b) nitrogen concentration retrieved with p-values < 0.01 (B5, B7, B8A, B12, and NDVI) and canopy nitrogen content retrieved with p-values < 0.05 (B2, B3, B4, B6, B7, B8, B11, B12, NDVI, and REP). The units of the Y-axes are the same as the units of the X-axis.

The differences between the best three models were not statistically significant. The stepwise linear regression was therefore selected for the further analyses because of its interpretabilty. It was retrained on the 120 parcels and its performance was further estimated through a leave-one-out cross-validation for the complete growing seasons 2016–2018, as shown in Fig. 3. Feature selection improved the results in all cases. From the leave-one-out calibration, the coefficients of determination of the stepwise linear regression were 0.55 for DM, 0.68 for NC and 0.44 for CNC.

For the DM retrieval (Fig. 3(b)), the best result is obtained when a p-value < 0.05 is applied for the variable selection, with an RMSE as low as 0.96 t/ha, corresponding to 16.0% of the range of measured DM values. The explanatory variables are B5, B6, B8A, B11, and the NDRE.

For the NC retrieval (Fig. 3(b)), a p-value threshold of 0.01 provided the best results, with a RMSE as low as 3.28 g/kg, and a normalized RMSE of 11.9%. The explanatory variables are B5, B7, B8A, B12 and the NDVI.

Finally, the CNC was retrieved most accurately with a p-value < 0.05 (Fig. 3(c)), resulting in a RMSE of 17.4 kg/ha, and a normalized RMSE of 14.5%, based on ten explanatory variables (B2, B3, B4, B6, B7, B8, B11, B12, NDVI, and REP).

### 3.3 Results of the validation

**Fig. 4:**
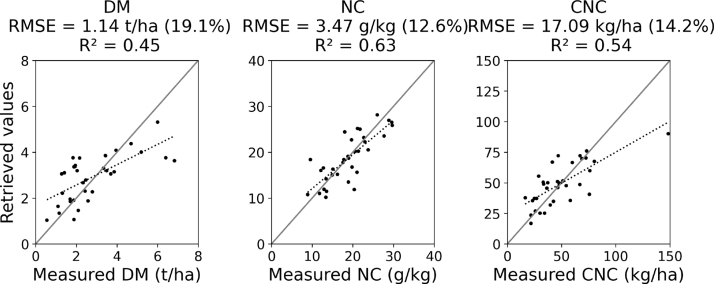
Validation results (n = 34) with the stepwise linear regression model using p-values <0.05. The Y-axis of each plot has the same unit as its corresponding X-axis.

The validation of the stepwise linear model on an independent dataset confirms the results obtained during the calibration step. The RMSE values were all in the expected range of the cross-validation results, with slightly better results for the CNC (17.09 vs. 17.4 kg/ha), and worse results for NC (1.14 vs. 0.96 g/kg) and for CNC (3.47 vs. 3.30 kg/ha). As observed on the calibration results, the main source of errors is the underestimation of large CNC and DM values (Fig. 4). The $R^{2}$ values between derived and measured values are 0.45, 0.63 and 0.54 for DM, NC and CNC, respectively.

**Fig. 5:**
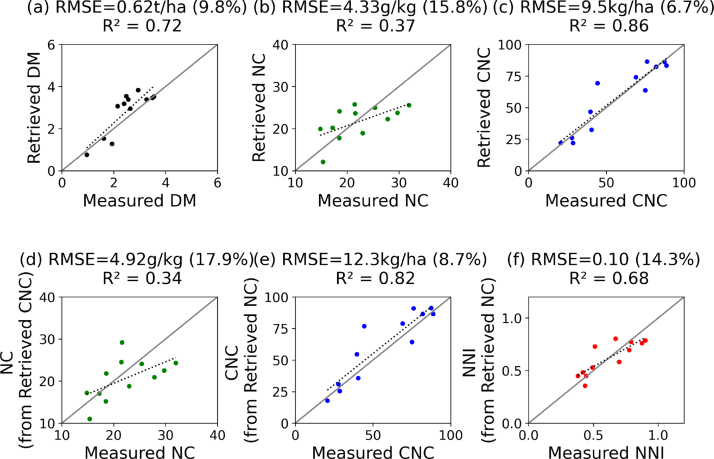
Validation of the retrieved (a) DM, (b) NC and (c) CNC, and of (d) NC, computed from the retrieved DM and CNC, (e) of CNC, computed from the retrieved DM and NC, and (f) NNI, computed from the retrieved DM and NC. The validation is based on the measurements (n = 12) of the 2020 field campaign.

The regression models calibrated on the years 2016–2018 were successfully validated using the field dataset of 2020 (Fig. 5(a), (b), and (c)). The DM was retrieved with high accuracy on the 12 sampled grasslands, resulting in a smaller RMSE than in the calibration (0.62 t/ha, 9.8%) and an improved correlation between the measured and retrieved values ($R^{2}$ = 0.72). The NC was retrieved with a larger RMSE of 4.33 g/kg (15.8%) and a poor $R^{2}$ (0.37). The CNC was retrieved with the highest accuracy, with an RMSE of 9.5 kg/ha (6.7%) and a $R^{2}$ of 0.86. These results suggest a good inter-annual transferability of the models. The models therefore show good inter-annual transferability for DM and CNC.

To test the indirect retrieval of NC and CNC, both were also computed based on the retrieved DM and the other expression of N (Fig. 5(d) and (e)). Based on the retrieved DM and CNC, the NC was estimated with an RMSE of 4.92 kg/ha (17.9%). Inversely, based on the retrieved DM and NC, the CNC was estimated with an RMSE of 12.3 kg/ha (8.7%). For both NC and CNC, the performances of indirect and direct estimations are very similar, but the indirect estimations result in larger errors.

Based on the retrieved DM and NC, the NNI was computed and validated as well (Fig. 5(f)). The RMSE is 0.10, the normalized RMSE is 14.3% and the $R^{2}$ is 0.68.

### 3.4 Large-scale application

**Fig. 8:**
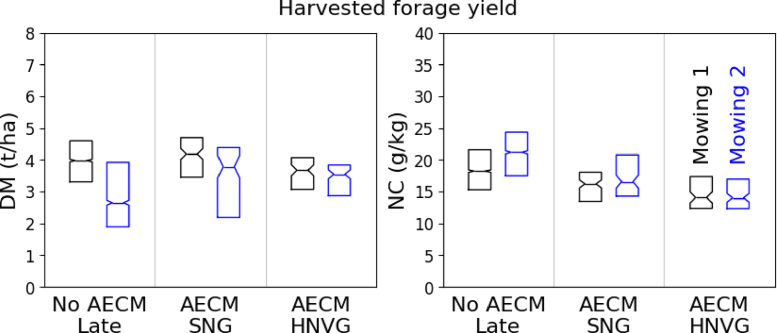
Boxplot distribution of DM and NC on the first mowing event in hay meadows (i) with a late first mowing event and no AECM, (ii) with AECM for semi-natural grassland (SNG) and (iii) with AECM for high nature value grassland (HNVG).

The DM and NC retrieval models were used to perform a spatially comprehensive analysis of the grassland status in the spring and of the harvested forage yield over the 2019 study area (Fig. 8). For these GUI analyzed accross different agroecological regions, the N status was estimated in terms of N concentration only — and not in terms of CNC, which is largely driven by the DM — since the results of the model calibration and validation showed good performances for the direct retrieval of NC.

#### 3.4.1 Status of the vegetation in spring

**Fig. 6:**
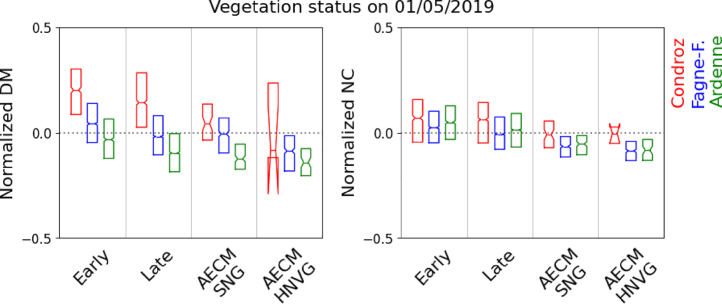
Boxplot distribution of normalized DM and NC on May 1st 2019 for different management practices per region. Management practices include pastures, hay meadows without AECM with an early or a late first mowing event, and hay meadows with AECM (semi-natural (SNG) and high nature value grasslands (HNVG)).

The DM and NC were estimated on the 1st of May 2019 for all pastures and all hay meadows with and without AECM not yet mown at that time. For each parcel, the retrieved biophysical variables were normalized by the overall average. The average normalized DM and NC values per agro-ecological region and per management practice are given in Table 2, Table 3, and Fig. 6.

**Table 2:**
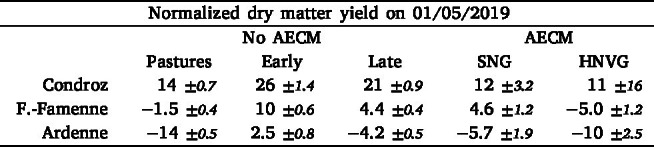
Average (with 95% confidence interval assuming a normal distribution) normalized retrieved DM on May 1st 2019 (in % of global mean) for different management practices per agroecological region. Management practices include pastures, hay meadows without AECM with an early or a late first mowing event, and hay meadows with AECM (semi-natural (SNG) and high nature value (HNVG)).

**Table 3:**
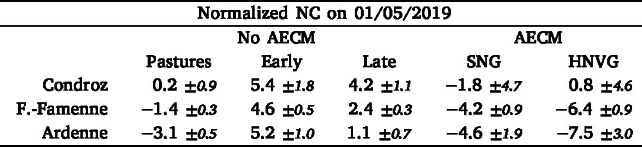
Average (with 95% confidence interval) normalized retrieved NC on May 1st 2019 (in % of global mean) for different management practices per agroecological region. Management practices include pastures, hay meadows without AECM with an early or a late first mowing event, and hay meadows with AECM (semi-natural (SNG) and high nature value (HNVG)).

The DM on the 1st of May is the highest in Condroz, followed by Fagne-Famenne, and Ardenne. In each region, the DM is the highest in hay meadows with an early first mowing event.

The DM then decreases, from no AECM hay meadows with a late mowing event, to SNG and finally HNVG, which have the lowest DM in all regions, but in Ardennes were they are second lowest to pastures. The differences in DM between SNG and no AECM hay meadows with a late first mowing event are relatively small, especially in Condroz and Ardenne. From the grasslands without AECM, pastures have the lowest DM in each region.

Across the three regions, the average NC is lower in hay meadows with AECM. Among hay meadows without AECM, the ones with an early first mowing have a slightly higher NC, although the difference with the ones with a late mowing event is small. The pastures have a lower average NC than the hay meadows. Among the AECM, the HNVG have the lowest NC in Fagne-Famenne and Ardenne. In Condroz, the number of AECM parcels is too small to observe statistically significant differences.

The variation in NC between regions is smaller and less consistent. The most significant difference is observed between the Condroz and the two other regions.

**Fig. 7:**
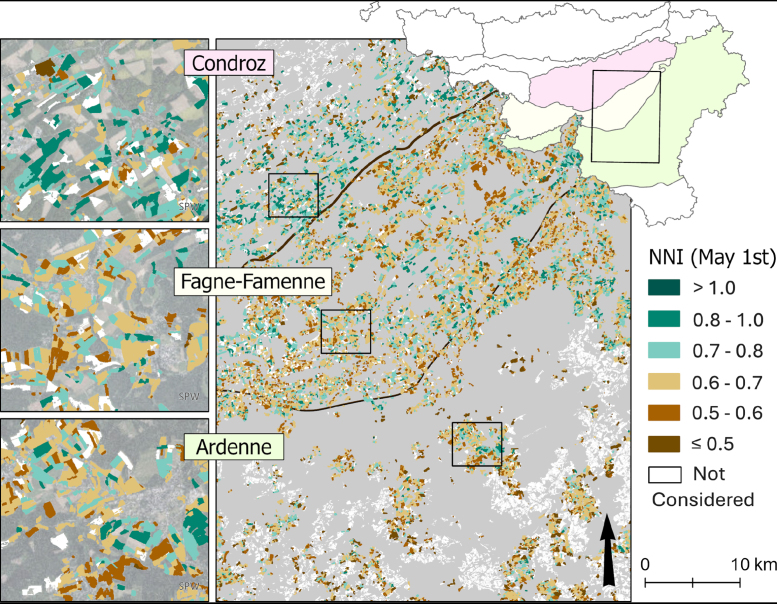
Nitrogen nutrition index computed from the retrieved DM and NC on the 1st of May 2019 across three agroecological regions of Wallonia. Some grasslands could not be considered, mostly because of a lack of S2 data due to cloud cover, or because they were already mown on the 1st of May. Orthophoto background for the zooms.

The NNI was computed from the DM and NC retrieved on May 1st, to map the nitrogen nutrition status of grasslands (Fig. 7). Some grasslands (14%) could not be considered, mostly because of a lack of S2 data due to cloud cover (13%), or because they were already mown on the 1st of May (1%).

SPW.

On this map, Condroz is characterized by a larger number of parcels with high NNI values, with 52% of the considered parcels above 0.7, compared to 29% in Fagne-Famenne and 25% in Ardenne. Very few parcels have NNI above 1, which would indicate potential overfertilization (15 in Condroz, 7 in Fagne-Famenne, and 5 in Ardenne). In each region, there are parcels with low NNI under 0.5, namely 7% in Condroz and Ardenne and 2% in Fagne-Famenne.

#### 3.4.2 Harvested forage yield

The average first mowing yields (DM and NC) of hay meadows with a late first mowing event (no AECM) and with AECM are given in Table 4 and Fig. 8. On the first mowing event, the DM is not significantly different in no AECM hay meadows and SNG (3.95 t/ha and 4.08 t/ha respectively). It is slightly lower in HNVG (3.59 t/ha). The second mowing DM is lower than the first mowing DM for each type of grassland and is the lowest in no AECM hay meadows (2.89 t/ha). It does not differ significantly between the two types of AECM (3.36 t/ha and 3.21 t/ha).

**Table 4:**
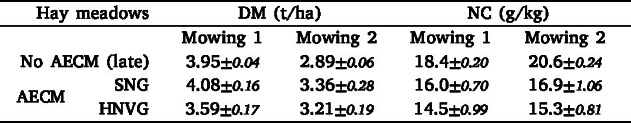
Average (with 95% confidence interval) retrieved dry matter yield (DM) and NC on the first and second mowing event in hay meadows with a late first mowing event (no AECM) and with AECM (semi-natural (SNG) and high nature value (HNVG)).

On both the first and second mowing event, the average NC is the highest in hay meadows without AECM (18.4 g/kg and 20.6 g/kg), followed by SNG (16.0 g/kg and 16.9 g/kg) and then HNVG (14.5 g/kg and 15.3 g/kg).

The first mowing yield (DM and NC) of hay meadows without AECM (with a late first mowing) was also averaged per region (Table 5). Unlike the DM on May 1st, the late first mowing DM is significantly the highest in Ardenne (4.29 t/ha). Fagne-Famenne and Condroz are characterized by lower late first mowing DM (3.66 t/ha and 3.57 t/ha). The NC is however higher in Condroz (19.5 g/kg) than in the two other regions (18.0 g/kg and 18.4 g/kg).

**Table 5:**
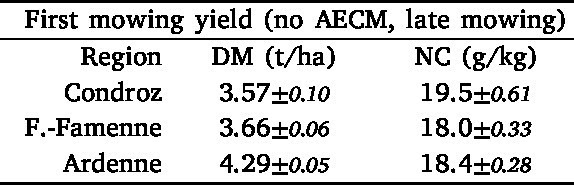
Average (with 95% confidence interval) retrieved dry matter yield (DM) and NC on the first mowing event of hay meadows with a late first mowing event (no AECM) per region.

## 4 Discussion

### 4.1 Biophysical variable retrieval

Previous studies on grassland biophysical variable retrieval have shown encouraging results, but were often conducted on small study areas (Ali et al., 2016b, Wang et al., 2019, Cisneros et al., 2020, Chiarito et al., 2021) and/or short periods (Zhang et al., 2015, Quan et al., 2017, Schwieder et al., 2020). In this study, 154 field measurements made during three growing seasons in 59 parcels, spread across three agroecological regions were used to calibrate the retrieval models.

For DM, the results of the calibration phase, covering the period of 2016–2018, are in line with — or lower than — those obtained in previous studies. Surprisingly, the DM retrieval model performed even better on the independent validation set collected in 2020 than on the validation dataset of the 2016–2018 period. This could be due to the scarcity of large DM values in the 2020 dataset, but it nonetheless shows a good temporal transferability of the model.

Our results on N content retrieval, based for the first time on actual satellite observations, corroborate findings of previous field spectroscopy studies (Cisneros et al., 2020, Pullanagari et al., 2021) and highlight the performances of S2-based large-scale grassland N monitoring.

While these previous studies retrieved N concentration, here we compare the retrieval of grassland N both in terms of concentration (i.e. NC) and of content per land area (i.e. CNC).

Although the opposite would be expected from a radiative transfer point of view, our results, and those of previous studies, show that NC can be directly related to S2 reflectances as well, even if the transferability of the NC model was lower than for the other two variables. Moreover, NC was estimated more accurately through the direct approach, than indirectly, based on retrieved DM and CNC (Fig. 5). While CNC was also better estimated directly than indirectly from DM and NC, the selected bands for the CNC regression model included most of the bands respectively selected for DM and NC models. These findings are quite expected because successive models cannot minimize the residual values unlike direct model based on the same features. Furthermore, it is striking that the independent validation results matched more with the theoretical expectations, with a significantly lower normalized RMSE for CNC than for NC retrieval. Overall, the S2 variables selected by stepwise multiple linear regression for each biophysical variable were found very consistent with known radiative transfer mechanisms. The red edge and NIR reflectances selected for DM respond to change in fresh biomass. The SWIR reflectance is more related to water content, lignin, cellulose, and senescent material (Hank et al., 2019). Since long, studies on grassland monitoring have successfully related above-ground biomass to these spectral domains and derived indices (Quan et al., 2017, Wang et al., 2019, Schwieder et al., 2020, Cisneros et al., 2020, Chiarito et al., 2021).

For the retrieval of NC and CNC, reflectances and indices derived from red edge and SWIR as well as the visible domain were retained. The red edge is a good indicator for chlorophyll content, which is in turn well related to N (Baret et al., 2007, Delloye et al., 2018, Hank et al., 2019). Previous studies have used SWIR reflectances to directly or indirectly retrieve crop N content (Herrmann et al., 2010, Berger et al., 2020b). Moreover, spectral signatures in the SWIR have been related to crop protein content, which carry a large part of their total N content (Berger et al., 2020a). Reflectances in the visible domain are most sensitive to pigments (including chlorophyll), which can explain the link with N (Hank et al., 2019).

The best empirical model was in our case the ordinary linear regression after stepwise feature selection, despite extensive grid search of the optimal hyperparameters of the other models. A much larger dataset would be necessary to fully exploit the potential of the more recent methods, but the results suggest that a linear relationship exists between the remotely sensed features and the biophysical variables studied in this paper. It is therefore not surprising that the linear regression performs better in this case because it minimizes the errors by design, as observed in other studies (Smith et al., 2013). On the other hand, all empirical models underestimated the large CNC values, which could be due to a saturation of the signal or the larger proportion of non photosynthetic vegetation (Xu et al., 2020).

Recent studies have increasingly used RTM for biophysical variable retrieval, as they are expected to offer better transferability for large-scale and multi-year applications and depend less on field measurements (Quan et al., 2017). Secondary variables such as DM and NC cannot be retrieved directly through deterministic approaches. In that case, RTMs are used to retrieve primary variables, such as LAI, chlorophyll content, or protein content, which can then be linked to DM, NC, or CNC (Weiss et al., 2020). In this study, we directly retrieved DM, NC, and CNC from S2 reflectances. Thanks to an extended training dataset, the empirical models showed good transferability across the years. The results for NC appear however less good in 2020, which could be due to a lesser transferability or to the small N range in the validation dataset.

In addition to the quantitative validation, the large-scale application of the models showed consistent trends in vegetation status and harvested forage yield in different types of grasslands across the study area. However, while the inter-annual range of the training dataset allowed a good temporal transferability of the models, the field measurements were less diverse in terms of grassland types and vegetation status as they were all made right before mowing — or grazing — events. In terms of absolute values, the early season DM seemed to be overestimated. Moreover, the average NC of pastures was surprisingly low compared to the other grasslands. This could be due to the heterogeneity of pastures, which contains refusal zones and trampled areas. Given the lower certainty of the absolute values, the early season DM and NC were normalized to perform a relative analysis. The intra-annual, spatial, and thematic transferability of these models should be further tested and would probably require additional training data.

The retrieval of NNI in particular should be considered with caution. The N dilution curve has been defined primarily for relatively intensive grasslands dominated by Gramineae species. It has also been shown to be valid for more diversified swards (Louarn et al., 2020). However, it is strongly recommended to limit the use of the N dilution curve to the period before flowering (Lemaire and Gastal, 2016), and the timing of the vegetative growth varies between regions and between vegetation species. Moreover, given the apparent overestimation of DM at that time, the NNI is probably overestimated as well. Locally, the retrieved NNI could, nevertheless, be used to identify outliers that have potentially received excessive fertilization.

Like in other optical remote sensing applications, a main limiting factor is the cloud coverage. In this study, cloud cover prevented the use of a large part of field measurements as no clear S2 acquisition could be associated with them. Moreover, in the large-scale application, the forage yield of 90% of the early mowing events could not be estimated and the nitrogen nutrition status of 13% of the parcels in the study area could not be assessed on the 1st of May, due to persistent cloud cover in the spring of 2019.

Finally, although a lot of information can be derived from S2’s multi-spectral data, a higher spectral resolution could probably improve the retrieval performance. Several studies using field spectrometry have linked grassland and crop biophysical variables to subtle spectral profile characteristics that can only be derived from hyperspectral data (Ramoelo et al., 2013, Hank et al., 2019, Pullanagari et al., 2021, Rubo and Zinkernagel, 2022). Recent and upcoming spaceborne high-resolution hyperspectral sensors (e.g. PRISMA, EnMAP, CHIME, EMIT) represent a great potential for grassland monitoring (Hank et al., 2019, Weiss et al., 2020) on medium to large parcels.

### 4.2 Grassland monitoring perspectives

The large field campaign of 2016–2018 was carried out to evaluate harvested forage yield and quality of grasslands with different AECM in comparison to other permanent grasslands. Such an intensive field campaign provides valuable information, but it is costly and time-consuming. The methods developed in this study allowed to extrapolate the field-based analysis, spatially and temporally.

First, our analysis showed that the grassland DM in the spring is driven by the pedo-climatic conditions of each agroecological region, Condroz being the most favorable, and Ardenne the least.

More strikingly, the regional boundaries appeared very clearly on the NNI map in Fig. 7. These differences in the vegetation status in spring between regions are probably mostly due to the variation in pedo-climatic conditions, and more specifically to temperature, which has an impact on the vegetation development and thereby on the regional management practices. The differences in management practices can in turn have an impact on the early season vegetation status and thereby further amplify regional trends.

Unlike the yield in spring, the average yield of late first mowing of hay meadows without AECM per region did not follow the expected pedo-climatic gradient. This could be due to regional tendencies in management practices, which are themselves influenced by climatic conditions. In Condroz and Fagne-Famenne, the most productive grasslands are likely to be mown early, while the grasslands with a late first mowing may be characterized by lower productivity. In addition, due to the omission of early mowing events by the Sen4CAP mowing detection method (De Vroey et al., 2022a), some of these late first mowing yields could actually be second mowing yields of grasslands with an early first mowing event, which would also explain the lower DM. In Ardenne, due to the climatic conditions, the vegetation cycle is slightly delayed compared to the other two regions, which means that most grasslands (more or less productive) are mown relatively late, which could explain the higher average late first mowing yield.

Secondly, we depicted the impacts of management practices on forage yield and quality in the spring and before harvest, allowing to link inputs and outputs in grassland use intensity. As expected, more intensively managed hay meadows (i.e. with an early first mowing) consistently showed higher average DM on the 1st of May, compared to the other management practices. These grasslands are more likely to have been fertilized at the start of the season to enhance their forage production in the spring. While the DM was impacted more by the pedo-climatic conditions, the NC seemed to be more driven by management practices and AECM.

Overall, the consistent matching obtained between the remotely sensed biophysical variables and mowing event detection with the respective grassland types defined by strict regulations highlights how well the GUI can be depicted through EO.

Remote sensing was used in previous studies to monitor grazing (De Vroey et al., 2022b, Awuah et al., 2020) or mowing (De Vroey et al., 2022a, Lobert et al., 2021, Holtgrave et al., 2023) practices, i.e. inputs of GUI. This study further showed the potential of remote sensing to monitor outputs of GUI and link them with inputs across different agro-pedo-climatic contexts.

The spatially explicit information on input and outputs of GUI obtained through this approach could be further linked with grassland biodiversity and ecological value. This could help bridging the knowledge gap on the complex relationship between inputs, outputs and outcomes of GUI and, finally, support sustainable agricultural policy.

## 5 Conclusions

In this study, grasslands DM and N content (NC and CNC) were retrieved from S2 spectral bands and indices through stepwise multiple linear regression, which outperformed more complex regression models such as random forest, support vector or neural network regressions. For each biophysical variable, the most explanatory variables were selected, and a model was calibrated, using a large field dataset, collected during 3 growing seasons. The models estimated DM and CNC on an independent validation dataset with normalized RMSE of 9.8% and 6.7% respectively, showing good temporal transferability. Finally, the models were integrated with classified management units and mowing detections to assess the impacts of management practices on the vegetation status in spring and harvested forage yield. These large-scale applications showed consistent results compared to the field measurements and allowed to highlight differences between agroecological regions.

In conclusion, on one hand the results of this study highlight the potential of Sentinel-2 for direct grassland biophysical variable retrieval. On the other hand, the approach developed in this study underlines current remote sensing capacities for large-scale and comprehensive GUI monitoring. More specifically, the integration of multiple EO-based methods can provide valuable knowledge on the relationship between inputs and outputs of GUI. Further efforts should be put into the development of more robust and scalable models and their integration into a multi-dimensional GUI monitoring framework.

## CRediT authorship contribution statement

**Mathilde De Vroey:** Writing – review & editing, Writing – original draft, Visualization, Validation, Methodology, Investigation, Formal analysis, Data curation, Conceptualization. **Julien Radoux:** Writing – review & editing, Writing – original draft, Supervision, Resources, Project administration, Methodology, Investigation, Funding acquisition, Conceptualization. **Arnaud Farinelle:** Writing – review & editing, Validation, Resources, Methodology. **Pierre Defourny:** Writing – review & editing, Supervision, Funding acquisition, Conceptualization.

## Declaration of competing interest

The authors declare that they have no known competing financial interests or personal relationships that could have appeared to influence the work reported in this paper.

## Data Availability

Data will be made available on request.
